# Prognostic value of CRP, PCT, and TNF-a, D-lactate, DAO, and endotoxin on survival of patients with severe craniocerebral injury

**DOI:** 10.5937/jomb0-54673

**Published:** 2025-09-05

**Authors:** Ming Gong, Han Peng

**Affiliations:** 1 Xingyi People's Hospital, Department of Neurological Surgery, Xingyi, Guizhou, 562400, China; 2 The Affiliated Hospital of Guizhou Medical University, Department of Neurological Surgery, Guiyang, Guizhou, 550002, China

**Keywords:** craniocerebral injury, hyperbaric oxygen, probiotics nutritional supplements, swallowing disorders, immune function, kraniocerebralne povrede, hiperbarični kiseonik, probiotski nutritivni dodaci, poremećaji gutanja, imunološka funkcija

## Abstract

**Background:**

Hyperbaric oxygen therapy is an important treatment method for early consciousness recovery and rehabilitation after brain trauma. In this study, we investigated the effects of hyperbaric oxygen combined with probiotics nutritional supplements (PNS) on patients with severe craniocerebral injury complicated with swallowing disorders in the Intensive care unit.

**Methods:**

Seventy-four patients with severe craniocerebral injury who received hyperbaric oxygen therapy in the ICU of our hospital from July 2020 to October 2023 were selected for retrospective analysis. Among them, 35 received conventional NS (control groups), and another 39 received PNS (research group). Inflammatory factors, T-lymphocyte subsets, and gastrointestinal mucosal function were compared between the two groups before and after treatment. In addition, patients' neurological function was assessed using the Glasgow Coma Scale (GCS), the Fugl-Meyer Assessment (FMA), and the National Institutes of Health Stroke Scale (NIHSS). Finally, the incidence of adverse effects between the two groups was counted.

## Abstract (sr)

**Uvod:**

Terapija hiperbaričnim kiseonikom predstavlja važan metod lečenja za rani oporavak svesti i rehabilitaciju nakon povrede mozga. U ovoj studiji ispitivali smo efekte hiperbaričnog kiseonika u kombinaciji sa probiotskim nutritivnim dodacima (PNS) kod pacijenata sa teškim kraniocerebralnim povredama koje su praćene poremećajima gutanja na odeljenju intenzivne nege.

**Metode:**

Za retrospektivnu analizu su odabrana 74 pacijenta sa teškim kraniocerebralnim povredama koji su od jula 2020. do oktobra 2023. godine primali terapiju hiperbaričnim kiseonikom na odeljenju intenzivne nege naše bol-nice. Među njima, 35 pacijenata je primilo konvencionalne nutritivne dodatke (kontrolna grupa), dok je 39 pacijenata primilo probiotske nutritivne dodatke (istraživačka grupa). Uporedili smo inflamatorne faktore, podskupove T-limfocita i funkciju gastrointestinalne sluzokože između dve grupe pre i nakon tretmana. Pored toga, neurološka funkcija pacinata je procenjivana pomoću Glazgovske skale kome (GCS), Fugl-Majerove skale procene (FMA) i Nacionalne skale instituta za zdravlje za moždani udar (NIHSS). Na kraju, za -beležena je učestalost neželjenih efekata u obe grupe.

## Introduction

Severe craniocerebral injury is a serious brain disease caused by direct or indirect violence to the head, resulting in craniocerebral tissue damage, with symptoms of consciousness disorders, headache, nausea, vomiting, epileptic seizure, limb paralysis, sensory disturbance, aphasia, and headache, etc., showing high rates of disability and mortality [1]. The incidence of craniocerebral injury has demonstrated a trend of increasing year by year, with a global incidence of 783.3 per 100,000 in 2022, accounting for 9–21% of traumas to all parts of the body [2]. In recent years, as medical technology advances, the mortality rate of patients with severe craniocerebral injuries has been significantly reduced; due to the complex condition, however, most of the patients suffer from swallowing disorders and insufficient nutrition intake, leading to increased risk of complications and poor prognosis [3] [4]. Therefore, patients should be treated more comprehensively to maximise their recovery.

Hyperbaric oxygen therapy is an important treatment method for early consciousness recovery andrehabilitation after brain trauma, which has been recognised by cerebral surgery experts [5]. Hyperbaric oxygen increases the oxygen partial pressure, oxygen content, and oxygen diffusion distance within the damaged brain tissue to reduce brain edema, promote neovascularisation, promote consciousness recovery and reduce sequelae [6]. In addition, due to the presence of swallowing disorders, patients are prone to hypoproteinemia and electrolyte disturbance caused by malnutrition, which increases the risk of infection [7]. Therefore, early supplementation with necessary nutrients allows for better patient recovery with sufficient energy, which is of great clinical significance in minimising complications and improving therapeutic efficacy [8].

However, there has been considerable clinical controversy in selecting nutrient solutions for enteral nutrition support. Traditional nutritional supplements (NS) are based on proteins, vitamins, and other substances, and their primary purpose is to provide essential nutrients for the body’s vital activities. Still, it is generally difficult to improve clinical efficacy or the prognosis of the patients further [9] [10]. We believe that adding the use of intestinal probiotics to traditional NS may be more helpful in the treatment of patients with dysphagia in road brain injury and improve their prognostic quality. However, there is still a lack of studies to confirm this.

Currently, hyperbaric oxygen combined with NS is common in patients with severe craniocerebral injury [11]. However, hyperbaric oxygen combined with probiotics nutritional supplements (PNS) is still uncommon in clinical practice, and we urgently need to validate the value of applying this therapeutic regimen. Here, hyperbaric oxygen combined with PNS was applied to patients with severe craniocerebral injury complicated with swallowing disorders in the Intensive care unit (ICU) for its effects on patient survival, thus providing an effective reference for clinical application in the future.

## Materials and methods

### Subjects

First, the sample size needed for this study was calculated using G*Power software. The parameters were set as follows: effect size=0.2, α=0.05, Power=0.8, and the output showed that a minimum of 34 study subjects were needed in each group. Seventy-four patients with severe craniocerebral injury who received hyperbaric oxygen therapy in the ICU of our hospital from July 2020 to October 2023 were selected for retrospective analysis. Clinical data of the two groups are shown in Table 1, with no significant differences between them (P>0.05). Among them, 35 received conventional NS (control groups), and another 39 received PNS (research group). The study was approved by the Ethics Committee of our hospital (Approval No: 2020-k201), and all the subjectssigned the informed consent form. All patients were diagnosed with severe craniocerebral injuries complicated with swallowing disorders by brain Computed Tomography (CT) or Magnetic Resonance Imaging (MRI), with a history of surgery and complete medical records. Patients with major tumour diseases, other severe trauma, serious dysfunction of essential organs, metabolic dysfunction, immune dysfunction, haemorrhage of the digestive tract or coagulation disorders were excluded.

**Table 1:** Comparison of clinical data.

Groups	n	Age	Male/female	Body Mass Index<br>(kg/m^2^)	Etiology		
					car accident	Fall	blows
control group	35	47.3±3.7	22(62.9)/13(37.1)	24.4±2.0	16(45.7)	5(14.3)	14(40.0)
research group	39	46.9±3.5	23(59.0)/16(41.0)	24.2±1.6	18(46.1)	6(15.4)	15(38.5)
t (χ^2^)		0.478	0.117	0.477	0.027		
P		0.634	0.733	0.635	0.987		

### Hyperbaric oxygen therapy

All patients received hyperbaric oxygen therapy in our hospital. The oxygen chamber pressure was set at 0.2 MPa, and the treatment lasted for 1 h, with a pressurisation time of 20 min, followed by a 10-minute rest period. The treatment session was conducted once a day, with 10 sessions constituting one course of treatment. Each course was followed by a one-week interval for four courses of treatment.

### Nutrition support

Control group: Patients received conventional NS (Fresenius Kabire Pharmaceuticals Ltd, H20183301, contains per 100 g: 10 g protein, 15 g fat, 61.2 g carbohydrates, 330 mg sodium ions, 500 mg potassium ions, 220mg phosphorus ions, 310 mg calcium ions, 1812 kJ energy), and a formulated total nutrient admixture was administered within 24 h after surgery, with a support standard of 20 kcal/kd/d. Research group: PNS [NS+Bifido bacterium triplex enterosoluble capsules (Jincheng Hais Pharmaceutical Co., Ltd, S19993065) 630 mg] was administered within 6 h after surgery. A silicone nasogastric tube was placed, and the enteral nutritional suspension was infused by continuous pump feeding. The drip rate and infusion volume of the nutritional suspension were strictly managed based on patient tolerance. Within 6–24 hours, the nutritional suspension was delivered through the tube at a uniform speed using a gravity pump to gradually allow the intestine to adapt to the nutrition support. From the next day, the infusion volume was increased by 200 mL daily, and full-dose standard-concentration total enteral nutrition was provided depending on the patient’s condition. The temperature of the nutritional suspension was maintained at 35–37°C to avoid abdominal pain, diarrhoea or other discomforts caused by low temperature. If necessary, intravenous infusions of electrolytes and trace elements were administered to the patients.

### Sample collection and testing

Before and after treatment, 3 mL of patients’ venous blood was collected in procoagulant tubes and centrifuged (3500 r/min, 10 min) after 30 min of standing at room temperature to obtain patients’ serum samples, which were stored in the refrigerator at -80°C. C-reactive protein (CRP), procalcitonin (PCT), and tumour necrosis factor-α (TNF-α) were detected by enzyme-linked immunosorbent assay (ELISA) using kits purchased from Wuhan Fearn Biotechnology Co. Ltd. T-lymphocyte subpopulations (CD3^+^, CD4^+^, and CD8^+^, and the ratio of CD4^+^/CD8^+^ would be calculated) were detected using a flow cytometer (Attune NxT, Thermo Fisher, USA). Gastrointestinal mucosal functions D-lactate, diamine oxidase (DAO), and endotoxin were detected using a fully automated biochemical analyser (BS-1000M, Myriad, China).

### Outcome measures

(1) Neurological functions: Before and after treatment, patient neurological function was assessed using the Glasgow Coma Scale (GCS) [12], Fugl-Meyer Assessment (FMA) [13], and the NationalInstitutes of Health Stroke Scale (NIHSS) [14]. The GCS, FMA, and NIHSS scores were inversely proportional to the severity of coma, motor ability, and neurological deficits, respectively (i.e., the lower the score, the more severe the condition). (2) Inflammatory reaction: CRP, PCT, and TNF-α. (3) Immune functions: T lymphocyte subsets. (4) Gastrointestinal mucosal functions: D-lactate, DAO, and endotoxin. (5) Safety: The incidence of complications during treatment and patient survival were statistically analysed.

### Statistical methods

Statistical analysis was performed using the SPSS 23.0. Enumeration data such as patient gender and incidence of complications were expressed as [n(%)], with the chi-square test for comparison. Measurement data such as ALB and GCS scores were expressed as (x̄ ± standard deviation), with the independent t-test for comparison between groups and the paired t-test for comparison within the same group. P<0.05 was considered statistically significant.

## Results

### Neurologic function after treatment was better in the research group than in the control group

Table 2 shows no difference in NIHSS, FMA, and GCS scores between the two groups before treatment (P>0.05). After treatment, the NIHSS score was (16.00±3.83) in the research group, which was lower than that before treatment and lower than that of the control group; the scores of FMA and GCS were (71.79±5.97) and (9.26±0.82), respectively, which were higher than that before treatment and higher than that of the control group (P<0.05).

**Table 2:** Comparison of neurological functions. Note: * indicates P<0.05 compared to before treatment. Glasgow Coma Scale, GCS; Fugl-Meyer Assessment, FMA; National Institutes of Health Stroke Scale, NIHSS.

Group	NIHSS		FMA		GCS	
	before treatment	after treatment	before treatment	after treatment	before treatment	after treatment
control group	46.71±4.34	59.60±5.51*	27.03±3.44	20.74±3.48*	6.83±0.38	9.26±0.82*
research group	46.18±3.94	71.79±5.97*	26.51±2.95	16.00±3.83*	6.85±0.59	11.13±0.80*
t	0.551	9.093	0.700	5.549	0.171	9.921
P	0.584	<0.001	0.486	<0.001	0.865	<0.001

### Inflammatory factors were lower in both treatment groups and lower in the research group

As shown in Table 3, after treatment, CRP, PCT, and TNF-α levels in both groups were lower than those before treatment, and these three levels were respectively (6.89±1.53) mg/L, (0.26±0.07) μg/L, and (31.40±5.18) μg/L in the research group, lower than those of the control group (P<0.05).

**Table 3:** Comparison of inflammatory reactions. Note: * indicates P<0.05 compared to before treatment. C-reactive protein, CRP; Procalcitonin, PCT; Tumor necrosis factor-α, TNF-α.

Group	CRP (g/L)		PCT (μg/L)		TNF-α (mg/L)	
	before treatment	after treatment	before treatment	after treatment	before treatment	after treatment
control group	19.21±4.19	8.26±1.66*	3.29±0.43	0.47±0.16*	50.73±7.72	35.40±6.22*
research group	19.21±4.27	6.89±1.53*	3.30±0.40	0.26±0.07*	50.77±8.39	31.40±5.18*
t	0.000	3.694	0.104	7.445	0.021	3.017
P	>0.999	<0.001	0.918	0.001	0.983	0.004

### Immunisation after treatment was better in the research group than in the control group

Table 4 shows no statistical difference between the two groups in T lymphocyte subsets before treatment (P>0.05). After treatment, CD3^+^, CD4^+^, and CD4^+^/CD8^+^ increased in both groups and were higher in the research group (P<0.05), while CD8^+^ decreased and was lower in the research group (P<0.05).

**Table 4:** Comparison of immune functions. Note: * indicates P<0.05 compared to before treatment.

Group	CD3^+^ (%)		CD4^+^ (%)		CD8^+^ (%)		CD4^+^/CD8^+^	
	before<br>treatment	after<br>treatment	before<br>treatment	after<br>treatment	before<br>treatment	after<br>treatment	before<br>treatment	after<br>treatment
control<br>group	54.01±2.58	65.15±5.18*	33.04±4.01	36.54±4.10*	30.48±3.90	26.93±2.92*	1.10±0.19	1.38±0.24*
research<br>group	53.53±3.84	72.39±5.97*	33.11±4.71	40.82±5.14*	30.47±3.54	23.45±3.96*	1.10±0.21	1.80±0.42*
t	0.624	5.542	0.068	3.930	0.012	4.261	0.000	5.201
P	0.535	<0.001	0.946	<0.001	0.991	<0.001	>0.999	<0.001

### Gastrointestinal mucosal functions after treatment were better in the research group than in the control group

As shown in Table 5, after treatment, D-lactate, DAO, and endotoxin were (1.63±0.26) mmol/L, (2.56±0.42) U/L, and (0.72±0.23) EU/L in the research group and (2.49±0.37) mmol/L, (3.07±0.37) U/L, and (1.36±0.23) EU/L in the control group, respectively. All these levels were lower than those before treatment in both groups and were lower in the research group compared to the control group (P<0.05).

**Table 5:** Comparison of gastrointestinal mucosal functions. Note: * indicates P<0.05 compared to before treatment. Diamine oxidase, DAO.

Group	D-lactate (mmol/L)		DAO (U/L)		Endotoxin (U/L)	
	before treatment	after treatment	before treatment	after treatment	before treatment	after treatment
control group	3.28±0.41	2.49±0.37*	5.03±0.41	3.07±0.37*	1.36±0.23*	1.36±0.23*
research group	3.41±0.48	1.63±0.26*	5.06±0.55	2.56±0.42*	2.14±0.42	0.72±0.23*
t	1.245	11.660	0.234	5.515	1.857	11.950
P	0.217	<0.001	0.793	<0.001	0.067	<0.001

### There was no difference in safety between the two groups

As shown in Table 6, complications were 10.26% in the research group and 28.57% in the control group. Comparing the two groups, the study group was lower than the control group (P<0.05). In addition, the mortality rate was 5.13% in the research group, which was also not different from that in the control group (P>0.05).

**Table 6:** Comparison of safety.

Group	n							
control group	35	4 (11.43)	2 (5.71)	2 (5.71)	1 (2.86)	1 (2.86)	28.57	8.57
research group	39	2 (5.13)	1 (2.56)	1 (2.56)	0 (0.00)	0 (0.00)	10.26	5.13
χ^2^							4.034	0.347
P							0.045	0.556

## Discussion

First, a comparison of improvement in neurological functions between the two groups revealed a lower NIHSS score but higher FMA and GCS scores in the research group, suggesting better neurological functions. Hyperbaric oxygen therapy can effectively alleviate trauma symptoms, promote patient recovery from coma, inhibit peroxidation, and enhance the antioxidant capacity of cells, effectively improving the recovery of nerve conduction and neurological functions [15]. We believe that hyperbaric oxygen therapy combined with PNS will further alleviate the patient’s hypermetabolic state and prevent metabolic disorders, thereby improving the conditions, promoting the recovery of neurological and motor functions, and improving their quality of life. Jiang HS et al. [16] showed that after craniocerebral injury, patients’ blood glucose increases rapidly, reducing glucose tolerance and enhancing protein breakdown. In the study of Yu J et al. [17], it was mentioned that PNS is richer in nutrients, in which Bifidobacterium triphylium is a better regulator of intestinal microecological balance, which can effectively inhibit the propagation ability of pathogenic bacteria, maintain the normal function of the intestinal tract, and promote the secretion of intestinal immune factors, thereby improving the body’s immune function. All of this help is more conducive to improving the patient’s state of health and promoting the recovery of neurological function.

The inflammatory reactions, immune functions, and gastrointestinal mucosal functions between the two groups were compared to verify our viewpoint. The results showed lower CRP, PCT, TNF-α, CD8^+^, DAO, and endotoxin levels. At the same time, CD3^+^, CD4^+^, CD4^+^/CD8^+^, and D-lactic acid were higher in the research group, demonstrating that hyperbaric oxygen combined with the PNS better improved inflammatory reactions, immune functions and gastrointestinal mucosal functions. Hyperbaric oxygen therapy effectively improves the aerobic metabolism of nerve cells, leading to the outward movement of calcium ions and the suppression of catecholamine secretion and release, preventing edema and inflammatory reactions [18]. Furthermore, hyperbaric oxygen therapy increases the patient’s blood oxygen levels and diffusion range, improving aerobic metabolism and maintaining circulation [19].

On the other hand, PNS rapidly and effectively replenishes the nutrients needed, and the ω-3 fattyacids contained promote the degradation of triglycerides and inhibit the synthesis and secretion of inflammatory mediators; additionally, the fatty acids, nucleotides, amino acids, and other components contained in the nutritional suspension maintain and repair local intestinal immunity with effect, thereby enhancing the overall immunity of the body [20]. The study by Zhang J et al. mentioned that bifidobacterium triplex can also effectively decompose the body’s sugar substances, make it produce lactic acid, regulate the intestinal balance, and then inhibit the generation of harmful substances in the intestinal tract to reduce the patient’s inflammatory response, and further promote the restoration of intestinal immune function [21]. Through the combined treatment of hyperbaric oxygen therapy and PNS, the accumulation of catecholamines was effectively inhibited, along with enhanced normal absorption of nutrients and improved blood flow of the gastrointestinal mucosa, promoting the recovery of gastrointestinal mucosal functions. In previous studies, hyperbaric oxygen combined with PNS has an excellent effect on improving the body functions of patients with cystitis radiation [22], which supports our viewpoint.

Finally, in the statistics of complications, it was seen that the study group had a lower complication rate, indicating that enteral PNS also helped improve patient safety. This is because Bifidobacterium trifidum can competitively inhibit the propagation of intestinal pathogenic bacteria, reduce the translocation of pathogenic bacteria in the intestinal tract, improve the barrier function of the gastrointestinal tract mucosa, and thus reduce the occurrence of gastrointestinal complications [23]. However, there was no difference in the prognostic mortality rate betweenthe two groups, but the small number of cases in this study does not exclude the possibility of chance. In the future, we will conduct a more comprehensive analysis based on this result to validate the findings of this study. In addition, we also needed to add clinical objective indicators to confirm further the full impact of hyperbaric oxygen combined with PNS in patients with craniocerebral injuries. Of course, optimising the formulation of PNS can be a daunting task. Subsequently, we should also conduct basic research to fully grasp the mechanism of probiotics’ effect on PNS in patients.

## Conclusion

In conclusion, hyperbaric oxygen combined with PNS improves the neurological functions of patients with severe craniocerebral injury complicated with swallowing disorders in ICU, inhibits their inflammatory reactions, and improves the immune functions and gastrointestinal mucous membrane functions, which better guarantees patient survival and prognosis.

## Dodatak

### Ethical approval

The study protocol was approved by the Humans Ethics Committee of The Affiliated Hospital of Guizhou Medical University (NO.2022081).

### Availability of data and materials

The data used to support the findings of this study are available from the corresponding author upon request.

### Funding

The authors declare that no funds, grants, or other support were received during the preparation of this manuscript.

### Acknowledgements

Not applicable.

### Conflict of interest statement

All the authors declare that they have no conflict of interest in this work.
